# The impact of storage buffer and storage conditions on fecal samples for bacteriophage infectivity and metavirome analyses

**DOI:** 10.1186/s40168-023-01632-9

**Published:** 2023-08-28

**Authors:** Xichuan Zhai, Josué L. Castro-Mejía, Alex Gobbi, Antonios Aslampaloglou, Witold Kot, Dennis S. Nielsen, Ling Deng

**Affiliations:** 1 Section of Microbiology and Fermentation, Department of Food Science, University of Copenhagen, Rolighedsvej 26, 1958 Frederiksberg C, Denmark; 2 Section of Microbial Ecology and Biotechnology, Department of Plant and Environmental Sciences, University of Copenhagen, Thorvaldsensvej 40, 1871 Frederiksberg C, Denmark

**Keywords:** Bacteriophage, Storage buffer, Storage condition, Infectivity, Genome recovery, Metavirome, Sequencing

## Abstract

**Background:**

There is an increasing interest in investigating the human gut virome for its influence on the gut bacterial community and its putative influence on the trajectory towards health or disease. Most gut virome studies are based on sequencing of stored fecal samples. However, relatively little is known about how conventional storage buffers and storage conditions affect the infectivity of bacteriophages and influence the downstream metavirome sequencing.

**Results:**

We demonstrate that the infectivity and genome recovery rate of different spiked bacteriophages (T4, c2 and Phi X174) are variable and highly dependent on storage buffers. Regardless of the storage temperature and timespan, all tested phages immediately lost 100% (DNA/RNA Shield) or more than 90% (StayRNA and RNAlater) of their infectivity. Generally, in SM buffer at 4 °C phage infectivity was preserved for up to 30 days and phage DNA integrity was maintained for up to 100 days. While in CANVAX, the most effective buffer, all spiked phage genomes were preserved for at least 100 days. Prolonged storage time (500 days) at – 80 °C impacted viral diversity differently in the different buffers. Samples stored in CANVAX or DNA/RNA Shield buffer had the least shifts in metavirome composition, after prolonged storage, but they yielded more contigs classified as “uncharacterised”. Moreover, in contrast to the SM buffer, these storage buffers yielded a higher fraction of bacterial DNA in metavirome-sequencing libraries. We demonstrated that the latter was due to inactivation of the DNases employed to remove extra-cellular DNA during virome extraction. The latter could be partly avoided by employing additional washing steps prior to virome extraction.

**Conclusion:**

Fecal sample storage buffers and storage conditions (time and temperature) strongly influence bacteriophage infectivity and viral composition as determined by plaque assay and metavirome sequencing. The choice of buffer had a larger effect than storage temperature and storage time on the quality of the viral sequences and analyses. Based on these results, we recommend storage of fecal virome samples at in SM buffer at 4 °C for the isolation of viruses and at – 80 °C for metagenomic applications if practically feasible (i.e., access to cold storage). For fecal samples stored in other buffers, samples should be cleared of these buffers before viral extraction and sequencing.

Video Abstract

**Supplementary Information:**

The online version contains supplementary material available at 10.1186/s40168-023-01632-9.

## Introduction

The mammalian gut is inhabited by a complex community of microbes (collectively referred to as the gut microbiome, GM), which is mainly composed of bacteria, with other biological entities such as bacteriophages (or phages; bacterial viruses) that are also important GM members. The GM plays important roles in host metabolism and immune system regulation and GM dysbiosis is linked to the development, and severity, of many diseases [1–3]. In the gut, phages are approximately as abundant as bacteria and they can influence bacterial diversity, abundance and function [4, 5]. Importantly, imbalances in the gut virome have been associated with diverse diseases including obesity, diabetes, alcoholic liver disease, necrotizing enterocolitis and malnutrition [1, 6–8]. Furthermore, the transfer of fecal virome communities from a healthy host to a diseased host can reverse the disease phenotype [6, 9–11]. Consequently, there is a strong interest in studying the gut virome to determine its role in the etiology of gut-related diseases. Crucially, the validity and reproducibility of such studies rely on the quality and stability of collected biological samples.

Inadequate storage of fecal samples can alter the distribution of specific taxa and yield biased sequencing results which can result in unreliable down-stream analyses [12–16]. Several studies employing sequencing-based microbiome characterization have reported that sample collection and storage methods have profound effects on the bacterial community profile. These studies emphasize the importance of employing optimal storage of fecal samples to yield the most accurate bacterial metagenomic analyses [13, 17–21]. However, to date little attention has been given to preservation of the gut virome. Currently, stabilization buffers are mainly tested for their suitability on the gut bacteria [14, 22, 23]; it is unclear to what extent these buffers can maintain bacteriophage infectivity during storage. Moreover, the effect of storage conditions on downstream fecal virome analysis has yet to be determined.

A major aim of this study was to investigate the effects of storage conditions (buffer, time and temperature) on the infectivity of three representative phages from the major phage families present in the gut. Furthermore, we determined the influence of storage conditions on meta-virome sequencing results.

## Results

**Fig. 1:**
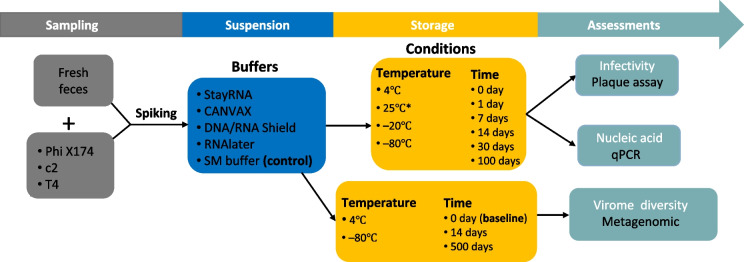
Workflow for phage storage assessments. * The phage-spiked fecal samples in buffers were also saved at 25 °C for 2 days then transferred to 4 °C and – 80 °C respectively for simulations of field sampling conditions where fecal samples cannot be stored under refrigeration or freezing conditions immediately

The effect of temperature (25 °C, 4 °C, − 20 °C, and – 80 °C) was investigated in 5 different storage buffers (StayRNA, CANVAX, DNA/RNA Shield, RNAlater, and SM buffer) with the aim of determining the effect of storage conditions on the infectivity of the phages T4, c2, and Phi X174 (up to 100 days of storage) as well as the overall virome composition and viral genome preservation (for up to 500 days) (Fig. 1).

### Buffers influence phage infectivity in different ways

**Fig. 2:**
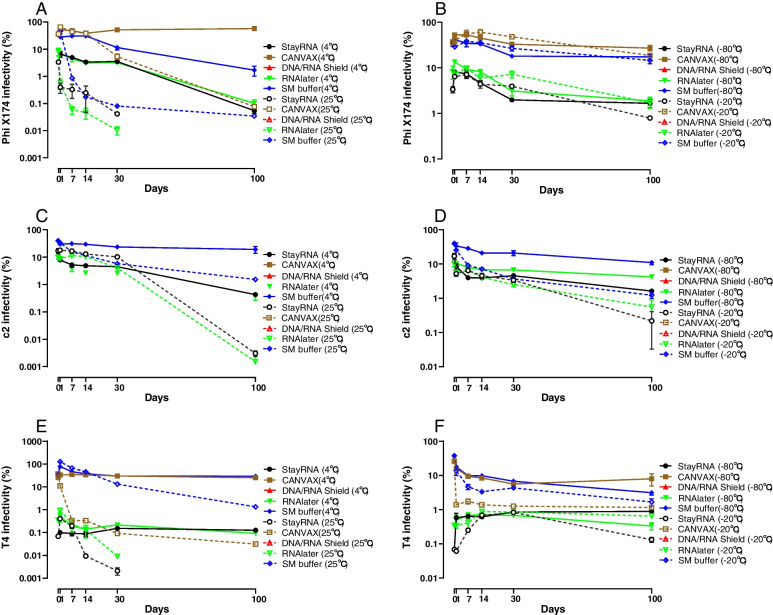
Effects of buffers and storage conditions (time and temperatures) on the infectivity of the spiked phages (Phi X174, c2, and T4) during 100-day storage. The percentages of phages recovered (*y*-axis, log scale) were determined by plaque assay at each different time point (*x*-axis). The error bars indicate the standard deviation with 3 replicates. No dots or lines indicate plaques were not detected. The left panels (**A**, **C**, **E**) represent the temperatures 25 °C (dotted lines) and 4 °C (solid lines) and the right panels (**B**, **D**, **F**) represent the temperatures 20 °C (dotted lines) and 80 °C (solid lines)

Initially, we determined the proportion of infectious phages that could be recovered immediately after spiking (day 0). As shown in Fig. 2 and Table S1, buffer DNA/RNA Shield inactivates all the tested phages rapidly (T4, c2, and Phi X174) regardless of storage temperature. The same result was observed for c2 in CANVAX. In contrast, various fractions of the tested phages remained infectious in StayRNA and RNAlater (0.07~17.0%) and SM buffer maintained the highest infectivity for all tested phages (34.6~39.7%).

Next, we determined the effect of different buffers on phage infectivity at different time points during 100 days of storage at various temperatures. At 25 °C, the infectivity of Phi X174 and T4 decreased rapidly during the first 30 days of storage in RNAlater and StayRNA, and no plaques were observed subsequently (Fig. 2A, E), while c2 remained infective after 100 days of storage (Fig. 2C). Colder temperature (4 °C) generally enhanced phage stability. CANVAX and SM buffer showed similar efficiency for both phage c2 and T4, but CANVAX maintained the highest infectivity for Phi X174 (Fig. 2A, C, and E). The infectivity of the Phi X174 and c2 showed substantial decreases (between 6- to 10-fold) in StayRNA and RNAlater after 30 days of storage, while T4 retained infectivity at relatively small fractions (0.09~0.13%) for up to 100 days of storage.

In general, all the phages tested could be plaqued during the storage period at – 20 °C and – 80 °C, if the infectivity was maintained at the time of spiking (Table S1, Fig. 2B, D, and F), though the plaque efficiency was quite different (from 0.13 to 60.8%). SM buffer and CANVAX were better than StayRNA and RNAlater at maintaining infectivity. Storage at − 80 °C was better for preserving infectivity than − 20 °C for c2 in SM buffer and T4 in CANVAX. Short term storage at 25 °C followed by transfer to the fridge (4 °C) or freezer (− 80 °C) resulted in a decline of infectivity of the tested phages comparable to the above single-stage temperature exposures (Figure S1A–C).

### Buffers preserve phage genomic content

**Fig. 3:**
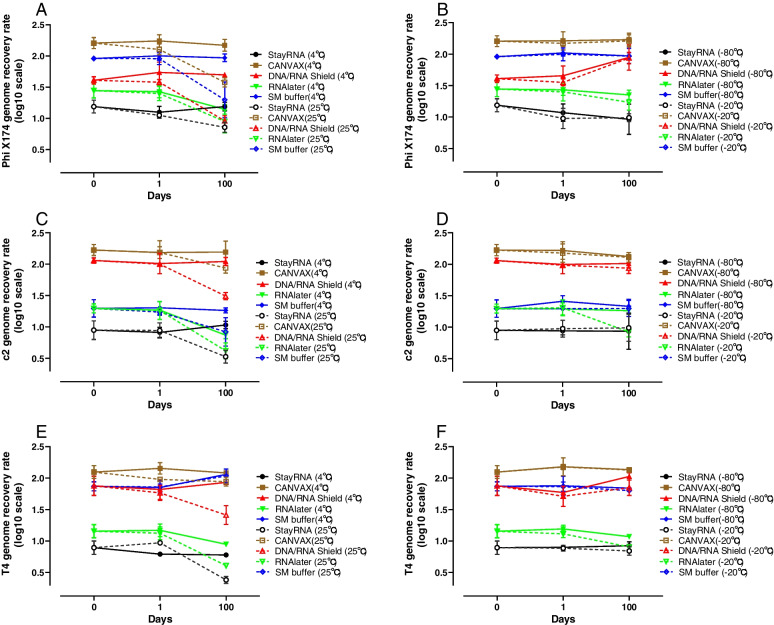
Effects of buffers and storage conditions (time and temperatures) on the genome recovery of the spiked phages (Phi X174, c2, and T4) over 100-day storage. The percentages of phage genomic recovery (*y*-axis, log10 scale) were determined by qPCR at each different time point (*x*-axis). The error bars indicate the standard deviation with 3 replicates. The left panels (**A**, **C**, **E**) represent the temperatures of 25 °C (dotted lines) and 4 °C (solid lines) and the right panels (**B**, **D**, **F**) represent the temperatures − 20 °C (dotted lines) and − 80 °C (solid lines)

Recovery of genomic content of the phages T4, c2, and Phi X174 spiked into fecal samples stored in the different buffers was determined by qPCR. All the spiked-in phage genomes were detectable in all the tested buffers to a larger or smaller degree after spiking (Fig. 3 and Table S1). CANVAX showed the best phage genome recovery rate for all phages, but also DNA/RNA Shield and SM buffer allowed good recovery (Table S1). All the tested buffers preserved DNA integrity for short periods of time (1 day) at all tested temperatures, but after long storage periods (100 days) at 25 °C a marked reduction in the fraction of genomes recovered was observed (Fig. 3A, C and E). When stored at lower temperatures (4 °C, − 20 °C, and – 80 °C) all the genomes were stable for up to 100 days. CANVAX showed the best capacity for preserving the phage genomes (Fig. 3B, D, and F), followed by SM buffer.

We also observed that initial temperature fluctuations during storage (25 °C for 2 days and then transferring to 4 °C or −80 °C) did not cause significant degradation of phage genomes, suggesting that samples obtained under field-like conditions without immediate access to cold storage are still usable for further metagenomic analysis (Figure S1D–F).

### Buffer and storage time affect gut virome diversity

Based on the experiments determining the infectivity and genomic recovery of the tested phages, we concluded that 4 °C and − 80 °C are favorable for phage storage. We then investigated how the viral community changed over time in response to different buffers and temperatures by comparing fecal viromes obtained after being stored for 0, 14, and 500 days under refrigeration (4 °C) and freezing (− 80 °C) temperatures, respectively (Fig. 1).

**Fig. 4:**
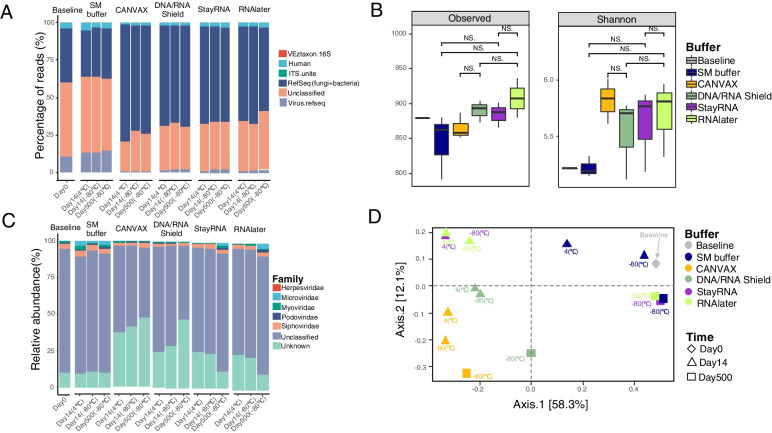
Effects of buffers and storage conditions (time and temperatures) on viral diversity. **A** Distribution of sequencing reads into the different taxonomic categories as viral, human, bacterial, fungi and unknown origin. To check the presence of non-viral DNA sequences, 50,000 random forward reads were evaluated according to their match to a range of viral, bacterial, and human reference genomes and protein databases. No reads (in 50,000 reads) matched the 18S rRNA gene sequences in all the samples. **B** The effect of buffers on the viral overall alpha diversity with the measurement of Observed taxa and Shannon diversity index. NS indicates not significant, one asterisk indicates a significant difference (*p* < 0.05, *t* test). **C** Representative taxonomic distribution (relative abundance) of the sequenced viromes. The relative distribution is described at the taxonomical level of the family. The taxonomy of contigs was determined by querying the viral contigs against a database containing taxon signature genes for virus orthologous group hosted at https://www.vogdb.org. The unclassified are the contigs that cannot be assigned to any known viral taxonomy at the family level, the unknown is the contigs that are related to “viral dark matter”. **D** Principal coordinates analysis (PCoA) plots of bray-Curtis distance matrices. PCoA was used to plot the beta diversity of viral-associated communities using the bray matrix. Different colors indicate different buffers, different shapes indicate different time points. For each axis, in square brackets, the percent of variation explained was reported

The buffers DNA/RNA Shield and CANVAX yielded the highest amount of nucleic acids, but with relatively low purity (Table S2), and the fraction of sequencing reads found to be of bacterial or fungal origin were much higher in CANVAX (> 70.5%) and DNA/RNA Shield (> 64.6%) stored samples compared to SM buffer (~ 30%). In line with this observation, a higher proportion of sequencing reads could be matched to viral databases when stored in SM buffer (9.0~14.3%) when compared with CANVAX (0.90~1.18%) and DNA/RNA Shield (1.74~4.45%) (Fig. 4A).

Although it appeared that the buffers had a strong influence on the distribution of viral and non-viral DNA, there were no significant differences amongst all the tested buffers in viral alpha diversity at the two tested storage temperatures (Fig. 4B). With long-term storage (500 days) a significant decrease in the viral Shannon diversity index was seen (*p* < 0.01), while the number of observed vOTUs did not change relative to the samples stored for 14 days (Figure S2A and B).

The majority of classified viruses were prokaryotic viruses (phages) belonging to the order of *Caudovirales* (e.g., *Siphoviridae*, *Myoviridae* and *Podoviridae*) and the order of *Petitvirales* (*Microviridae*), but most of the identified viruses cannot be classified at the family level (Fig. 4C). The “unknown” category was larger when samples were stored in CANVAX (37~47%) and DNA/RNA Shield (24~47%) compared to SM buffer (< 10%) (Fig. 4C and Table S3). Further, *Caudovirales* constituted a higher fraction of the contigs when stored in SM buffer (~ 60%) than all the other buffers (Table S3). *Siphoviridae* was the most abundant identified family in the baseline (in SM buffer, day 0) and SM buffer samples, but was less abundant in CANVAX (*p* = 0.002, Wilcox test) and DNA/RNA Shield (*p* = 0.004, Wilcox test) regardless of storage temperature and time.

Bray-Curtis distance-based metrics showed clear differences among the viromes stored in different buffers (Fig. 4D, PERMANOVA, *p* = 0.019), with samples stored in CANVAX clustering close to samples stored in DNA/RNA Shield, while the StayRNA samples clustered with samples stored in RNAlater. Short-term storage (14 days) at refrigeration (4 °C) and freezing (− 80 °C) temperatures did not strongly affect the viral distribution (PERMANOVA, *p* = 0.225), but long-term storage (500 days) led to a distinct shift in viral distribution (PERMANOVA, *p* = 0.014), especially for the viral community in StayRNA, RNAlater, and SM buffer. Notably, samples stored short-term in SM buffer at – 80 °C clustered more closely to the baseline (day 0) sample.

### Buffers induce non-viral sequencing bias

**Fig. 5:**
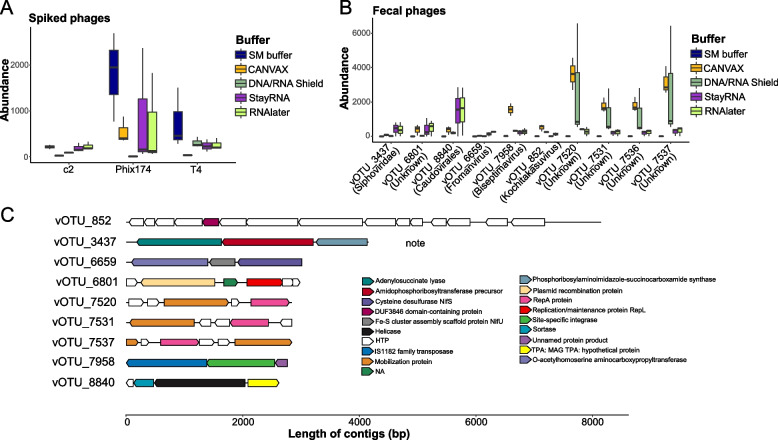
The preservation buffers lead to “sneaker contigs”. **A** Abundance of spiked phages (Phi X174, c2, and T4). **B** Abundances of representative contigs are annotated at different taxonomy levels, the selected contigs are the contigs that have high abundance in the buffers other than SM buffer. **C** Genomic maps of the open reading frames (ORFs) which are predicted by prodigal and then annotated by blast to the NCBI protein database; the best hits were used for the annotation. Different colors indicate different annotated proteins, directional boxes indicate ORFs in the respective orientation. NA: not assigned, HTP: hypothetical protein

The abundance of spiked phages (T4, c2, and Phi X174) recovered from the SM buffer by sequencing was higher than that found in the other tested buffers, especially for the ssDNA phage Phi X174 (Fig. 5A). However, we found that samples in nearly all the buffers, except for the SM buffer, have high levels of bacterial sequences, as there was very low abundance (close to zero) in SM buffer but high abundance in StayRNA, RNAlater, and CANVAX of contigs that encoded a lot of hypothetical proteins (HTP) from bacterial genomes or draft genomes (Fig. 5B, C). We determined these sequences to be “sneaker contigs” that are derived from bacterial DNA not completely removed during virome purification (Fig. 5C, Figure S3).

**Fig. 6:**
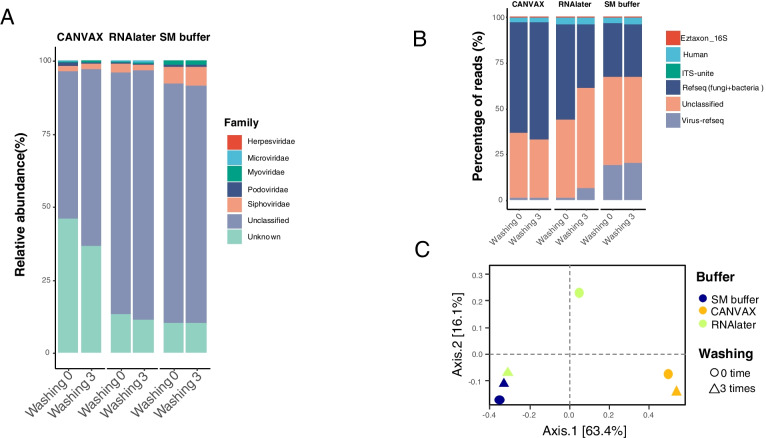
Washing improves virome quality in RNAlater. **A** Representative taxonomic distribution (relative abundance) of the sequenced viromes after 3 washes with SM buffer. **B** Distribution of sequencing reads into the different taxonomic categories as viral, human, bacterial, fungi and unknown origin after 3 washes with SM buffer. **C** Principal coordinates analysis (PCoA) plots of Bray-Curtis distance matrices. The data analysis procedure is described in Fig. 4

The virome purification protocol contains a nuclease treatment step to remove environmental DNA before the viral capsids are lysed and viral DNA/RNA purified. We hypothesized that the addition of RNAlater and CANVAX buffers may inhibit the nuclease activity. To test this, we spiked exogenous viral DNA into the two buffers and treated with the nuclease while SM buffer was used as a control. We found that both RNAlater and CANVAX completely inactivated the nuclease activity and therefore left extracelluar DNA undigested (Figure S4A). Repeated washing steps with SM-buffer allowed us to counteract this inhibition in RNAlater samples, but not CANVAX (Figure S4B). In line with removing inhibition from the RNAlater samples, our metavirome sequencing result also showed the RNAlater samples treated with an extra washing step had a similar virome diversity to that in SM buffer (Fig. 6C), while the virome sequencing quality of CANVAX-stored samples was not improved (Fig. 6A, B).

## Discussion

Storage conditions for fecal samples saved for subsequent virus/phage isolation or metavirome sequencing have not been thoroughly investigated previously. Here, we studied the impact of storage conditions (buffer, temperature, time) on the infectivity of spiked bacteriophages and the overall fecal viral community composition.

One of the most important prerequisites for successful viral application is its infectivity. Here we found the infectivity of the different bacteriophages was quite variable and appeared to be highly dependent on storage buffer and temperature. Our results showed that phages immediately lost most of their infectivity (> 60%) after spiking (Fig. 2 and Table S1), and all the tested phages with non-enveloped structures completely lost their infectivity in DNA/RNA Shield and the same for the long-tailed phage c2 phage in CANVAX buffer. This may result from the low pH values of these buffers (Table S2), as the isoelectric point (IS) affects the net surface charge of the coat protein of non-enveloped viruses and impairs their ability to attach to their host [24–26]. Another explanation may be that the high osmotic stress in both DNA/RNA Shield and CANVAX lead to osmotic shock and possibly cause the inactivation of bacteriophages [27]. Consistent with previous studies, our results showed that the commonly used SM buffer overall maintained phage infectivity well [28–33], possibly due to its neutral pH and that buffer ions interact with capsids and stabilize the protein structures [34].

Several 16S rRNA gene-based studies have shown that the use of preservation buffers (such as RNAlater) for protecting the bacteria at room temperature (RT) were not always effective [22, 23]. This is also relevant to phage storage at unfavorable temperatures which can lead to a faster degradation of the protein structures that constitute the capsid [35], which is consistent with our results showing that phages stored at 25 °C lose their infectivity faster than when stored at lower temperatures. At − 20 °C the tailed phages (c2 and T4) showed a faster decrease in infectivity than the non-tailed phage Phi X174, probably due to slow freezing that can induce more crystals that damage the fragile phage tail structure [36]. Hence, it is important to ensure fast freezing of the samples if they are intended for phage isolation in virome studies [37, 38]. In many situations storage at 4 °C (or in wet ice at 0 °C) is convenient, and phage infectivity in SM buffer or CANVAX at 4 °C is better preserved than at − 20 and − 80 °C.

Importantly, as long as the phages were stabilized in buffers, the storage time and temperature (except for 25 °C for a long time) do not affect the genome recovery efficiency as determined by qPCR (Fig. 3). This suggests that the overall phage structure was preserved and the phage DNA was intact [32]. After meta-virome sequencing Bray–Curtis dissimilarity metrics showed that samples stored in DNA/RNA Shield grouped with CANVAX, and RNAlater grouped with StayRNA, respectively which suggested that they have similar storage functions (Fig. 4). Except for a few low coverage regions of Phi X174 and T4 in CANVAX and DNA/RNA Shield (Table S6), all the sequences of the spiked-in phages could be detected, indicating all the buffers have preserved the viral DNA in the viral particles. The recovered relative abundance of spiked phage Phi X174 is higher than c2 and T4 in the metavirome analysis even though the same amount of spiked-in phage was used for all three phages (Fig. 5A). This may be due to the Phi X174 genome being easier to extract or its circular ssDNA genome is preferentially amplified by MDA or both [39, 40]. To minimize potential bias due to MDA-based overamplification, we reduced the reaction time to ½ hour instead of the 2 h recommended by the manufacturer [41]. Furthermore, even though MDA potentially leads to over-amplification of ssDNA phages like Phi X174, conclusions based on the overall metavirome still holds, as underlined by the fact that the largest meta-analysis of human gut viromics data did not find any significant differences between MDA amplified and unamplified viromes [42]. Further, using plaque assays a recent infant gut virome study showed that the relationship between *Escherichia coli* attacking dsDNA viruses quantified by plaque assays and by MDA-amplified metavirome was still linear [41]. The low bacterial/eukaryotic DNA contamination from the samples stored in SM buffer is consistent with our previous observations [32, 33]. Both CANVAX and DNA/RNA Shield can preserve more nucleic acids from the samples and yielded higher DNA concentrations (Table S1) but with a lower purity and a higher degree of bacterial and eukaryotic DNA contamination (Fig. 4).

The elimination of non-viral nucleic acids during virome extraction is vital. However, all the tested storage buffers except SM buffer can lead to higher bacterial contamination of the viromes due to their inhibiting nuclease activity used to remove planktonic bacteria during virome extraction (Fig. 6 and Figure S4). Importantly, the inhibition of the nuclease activity in RNAlater can be relieved by SM buffer washing, probably by removing the chemicals that cause deactivation, but this did not work for CANVAX stored samples (Fig. 6 and Figure S4). The SM buffer washing step can be used for both StayRNA and RNAlater if samples are already stored in these two buffers, but deeper sequencing and more careful viral contig verification may be needed for samples saved in CANVAX and DNA/RNA Shield.

## Conclusion

Our data shows the importance of storage conditions for virome studies. We found that phage infectivity was maintained most effectively in SM buffer at 4 °C. However, when there is no need to isolate of active phages from samples, using SM buffer, and storage at − 80 °C, leads to more stable and less potential contamination for metavirome studies. If the StayRNA or RNAlater buffer are used, intensive sample washing with SM buffer before the nuclease treatment should be performed.

## Materials and methods

### Phage propagation and plaque assays

Three phages from different families, namely phage T4 (*Myoviridae*), phage c2 (*Siphoviridae*), and phage Phi X174 (*Microviridae*) (Table S4) were produced in this study. The host of phage T4, *Escherichia coli* DSM 613, was grown in LB broth (Merck, Kenilworth, NJ, USA) at 37 °C with shaking at 225 rpm. Phage c2’s host, *Lactococcus lactis* MG1363, was grown in M17 broth (Merck, Kenilworth, NJ, USA) supplemented with 5 mM CaCl_2_ at 30°C without shaking. The host of Phi X174, *Escherichia coli* ATCC 13706 was grown in BHI broth (Merck, Kenilworth, NJ, USA) containing 10 mM CaCl_2_ and MgCl_2_ at 37 °C and shaking at 225 rpm. For phage propagation, phages were incubated with their respective host bacteria overnight and then subjected to centrifugation at 5000×*g* for 30 min at 4 °C to remove cell debris. The phage stocks were prepared using a 0.45 µm filter and stored at 4 °C. The infectivity of the phages in the filtrates was enumerated by plaque assay as described below.

### Preparation of phage spiked fecal samples

A fresh fecal sample was donated by an anonymous healthy adult donor and mixed thoroughly with 5 different preservation buffers, namely StayRNA (A&A Biotechnology, Gdynia, Poland), CANVAX (Canvax Biotech, Córdoba, Spain), DNA/RNA Shield (Zymo Research, Irvine, CA, USA), RNAlater (Sigma-Aldrich), and SM (Sodium chloride/Magnesium sulfate) buffer (lab preparation, 200 mM NaCl, 10 mM MgSO_4_, 50 mM Tris-HCl (1 M, pH 7.5)). Then, the fecal suspensions were spiked with different volumes of phages to a final concentration of 3 × 10^5^ plaque-forming units per milliliter (3 × 10^5^ PFU/mL) of each phage and mixed gently to form the spiked fecal samples.

### Storage at different temperatures and times

The above prepared spiked fecal samples were stored at different temperatures (room temperature at 25 °C, refrigeration at 4 °C, freezing at − 20 °C and − 80 °C) and the infectivity of phages was detected at different time points (0, 1, 7, 14, 30, and 100 days) by plaque assays. Briefly, 100 μL of the spiked fecal sample was centrifuged at 12,000 rpm for 10 min and 10 μL of the supernatant with different dilutions containing the phages (c2, Phi X174, and T4) was mixed with 200 μL of their respective overnight cultured hosts *Lactococcusl lactis* MG1363, *Escherichia coli* ATTC 13706B1 and *Escherichia coli* DSM 613 and left to settle for 10 min at room temperature. Five mL of media containing 0.5% agarose pre-warmed at 40 °C was mixed with the phage sample and bacterial culture and poured to the top of a pre-warmed agar plate (1.5%). The double-layer plates were first solidified at room temperature and then incubated overnight at the corresponding growth temperature of the bacterial host. On the next day, the phage plaques were counted, and PFU/mL was calculated. The infectivity of the phage was calculated using the following formula:

Phage infectivity (%) = Plaque amounts/Dilution factors/Added volume of diluted phage/The initial concentration of phage × 100. The genome recovery rate of the spiked phages was determined by qPCR at 0, 1, and 100 days. Furthermore, we simulated a field sampling condition where fecal samples cannot be stored under refrigeration or freezing conditions immediately. For this, we kept the spiked fecal samples at 25 °C for 2 days and then transferred them to 4 °C and – 80 °C for 14 days for the infectivity and genome recovery study, respectively (Fig. 1). We also stored the same batch of spiked fecal samples for the virome diversity study, where a baseline sample was prepared at day 0, and samples stored at 4 °C and – 80 °C for 14 days, − 80 °C for 500 days were prepared for metavirome sequencing.

### Purification and pretreatment of virome DNA

Phage isolation and purification were carried out according to our previous method with minor modifications [33]. Briefly, the Centriprep 50K was replaced by Centrisart^®^ I centrifugal ultrafiltration unit (MWCO 100 kDa, Sartorius Stedim Biotech GmbH) and the enrichment step was done at 2500×*g* for 30 min at 4 °C or 25 °C. Extra centrifugation times were applied for some difficult samples (Table S2). QIAmp viral RNA mini kit (Qiagen, Hilden, Germany) was used for the extraction of viral DNA/RNA from the concentrated virome solution. The extracted nucleic acids were amplified by Multiple Displacement Amplification (MDA) with the Genomephi V3 kit (GE Healthcare Life Science, Marlborough, MA, USA), and the amplification time was done at 30 °C for 30 min. Finally, the amplified DNA was cleaned with a Genomic DNA Clean & Concentrator^TM^ kit (Zymo Research, Irvine, CA, USA).

### Phage quantification by quantitative real-time PCR (qPCR)

The DNA from phage T4, c2, and Phi X174 was quantified by real-time qPCR using SYBR Green Master Mix (Roche, Basel, Switzerland) on CFX96 Touch Real-Time PCR Detection System (Bio-Rad, Hercules, CA, USA). Ten μM of forward and reverse primers targeting the specific T4, c2 and Phi X174 genome were added to 20 μL reactions, which were run using the following setup: initial stage at 50 °C for 2 min, hot start at 95 °C for 10 min, followed by 40 cycles of (i) 95 °C for 15 s, (ii) 55 °C for 20 s, and (iii) 60°C for 40 s [33]. Serial 10-time dilutions of phages (T4, c2, and Phi X174) genomic DNA were used to generate standard curves. After the qPCR amplification, a melting curve analysis (95 °C for 15 s, 60 °C for 60 s, 95 °C for 30 s and 60 °C for 15 s) was performed. Each reaction was performed in duplicates and the designed primers for specific phages and targeted positions were listed in Table S5.

To investigate the activity of universal nuclease in the buffer of RNAlater, CANVAX and SM buffer, the exogenous DNA was spiked into the above buffers and carried out through the same extraction procedure. Fecal samples kept in the above buffers were enriched and washed with SM buffer up to 5 times to verify if washing improved fecal virome quality.

### Metavirome sequencing and data pre-processing

The concentration of the MDA amplified and cleaned DNA was measured by Qubit dsDNA HS Assay Kit (ThermoFisher Scientific, Waltham, MA, USA). The library was constructed using the Nextera XT kit (Illumina, San Diego, CA, USA) and purified by AMPure XP beads according to the manufacturer’s protocol. Constructed libraries were sequenced using 2 × 150 bp paired-end settings on an Illumina NextSeq550 platform.

The average sequencing depth for the metavirome was 3,646,735 reads/sample (Table S6, min. 661,636 reads and max. 6,529,708 reads). The raw reads were trimmed from adaptors and barcodes and the high quality sequences (> 95% quality) using Trimmomatic v0.35 [43], with a minimum size of 50nt were retained for further analysis. High-quality reads were de-duplicated and checked for the presence of Phi X174 using BBMap (bbduk.sh) [44]. Virus-like particle-derived DNA sequences were subjected to within-sample de-novo assembly-only using Spades v3.13.1 [45]; contigs with a minimum length of 2200 nt, were retained. Contigs generated from all samples were pooled and de-duplicated at 90% identity using BBMap (dedupe.sh) [44]. Prediction of viral contigs/genomes was carried out using VirSorter2 [46] (“full” categories | dsDNAphage, ssDNA, RNA, Lavidaviridae, nucleocytoplasmic large DNA viruses (NCLDV) | viral quality ≥ 0.66), vibrant [47] (High-quality | Complete), and checkv [48] (High-quality | Complete). Taxonomy was inferred by blasting the predicted viral ORF against viral orthologous groups (vog206) [49] and for each viral contig the annotated proteins/genes were subjected to voting-consensus Lowest Common Ancestor (LCA) system (winner-gets-it-all) based on a minimum *e* value of 10e−5. Following assembly, quality control, and annotations, reads from all samples were mapped against the viral (high-quality) contigs (vOTUs) using bowtie2 [50] and a contingency-table of reads per Kbp of contig sequence per million reads sample (RPKM) was generated, here defined as vOTU-table. Code describing this pipeline can be accessed in github: github.com/jcame/virome_analysis-FOOD.

### Data analysis

The infectivity and genome recovery were visualized by GraphPad Prism (v8.0.1) or R software (v4.1.2). Analysis of viral community α- and β-diversity were performed using packages Phyloseq (v1. 36.0) [51] and Vegan (v2.5.6) in R. For α-diversity analysis, all the indices were calculated with *t* test using packages ggsignif (v0.6.3). Bray-Curtis distance metrics were calculated for β-diversity analysis and unconstrained ordination was performed using principal coordinate analysis (PCoA). R package gggenomes was used to visualize the functional genes of annotated viral contigs [52].

### Supplementary Information

**Additional file 1: Figure S1.** Effects of buffers and temperatures on the infectivity of spiked phages (A-C) and phage genomes (D-F). The percentages of phage activity (y-axis) or phage genomic recovery (y-axis, log10 scale) were determined by plaque assay at each different time point (x-axis) or qPCR. The error bars indicate the standard deviation with 3 replicates. Direct and indirect storage conditions were tested: in the direct storage condition, phage-spiked fecal samples were stored directly at 4 or − 80°C. In the indirect storage condition, phage-spiked fecal samples were first stored at 25°C for two days and then transferred to 4 or − 80°C, as described in the methods section. **Figure S2.** The effects of temperatures (A) and time (B) on the viral overall-alpha diversity with the measurement of Observed and Shannon index. NS indicates not significant, and two asterisks indicate a highly significant difference (*p* < 0.01, t-test). **Figure S3.** Representative of “sneaker contigs”. (A) The abundances of representative contigs annotated at different taxonomy levels, the selected contigs are based on contigs at high abundance compared to those in SM buffer (where the abundance was close to 0). (B) Genomic maps of the open reading frames (ORFs) which are predicted by prodigal and then annotated by blast to the NCBI protein database; the best hits were used to visualize the functional regions of these contigs. Different colors indicate different annotated proteins, directional boxes indicate ORFs in the respective orientation. NA: not assigned, HTP: hypothetical protein. **Figure S4.** Universal nuclease activity tests in the selected storage buffers (RNAlater, CANVAX and SM buffer). The selected buffers were spiked with exogenous DNA and then the activity of universal nuclease was tested. (A) The residues of exogenous DNA after 10 and 30 min treatments with universal nuclease in the selected buffers.  (B) The residues of exogenous DNA in the selected buffers with a different number of washes with SM buffer. The error bars indicate the standard deviation with 3 replicates. **Table S1.** Plaque assay and qPCR-based phage recovery rate (%) after spiking phages (T4, c2 and Phi X174) in fresh fecal with different buffers (day0). **Table S2.** Differences in the fecal virome isolation process with different buffers. **Table S3.** Relative abundance (%) of viral composition at order level. **Table S4.** Phages and their respective host bacteria in the present study. **Table S5.** Primers and targeted position for T4, c2 and Phi X174 genomes. **Table S6.** The number of reads and coverage of spiked phages (T4, c2 and Phi X174) to their respective reference genomes. The coverage rate was calculated by Bowtie2.

## Data Availability

The viral metagenome sequences raw data produced in this study is available through the NCBI Sequence Read Archive under BioProject accession number PRJNA926386. Code for virome pre-analysis is available from github: github.com/jcame/virome_analysis-FOOD.

## Abbreviations

GM: Gut microbiome
NA: Not assigned
RT: Room temperature
MDA: Multiple displacement amplification
ssDNA: Single-stranded DNA
dsDNA: Double-stranded DNA
PCoA: Principal coordinates analysis
vOTU: Viral-operational taxonomic unit
ORFs: Open reading frames
NCLDV: Nucleocytoplasmic large DNA viruses
LCA: Lowest Common Ancestor
SGBs: Species-level genome bins
PERMANOVA: Permutational multivariate analysis of variance
HTP: Hypothetical proteins
