# Supplementary material for: The impact of storage buffer and storage conditions on fecal samples for bacteriophage infectivity and metavirome analyses
Source: Microbiome. 2023 Aug 28;11:193. doi: 10.1186/s40168-023-01632-9 (PMC10463696; doi:10.1186/s40168-023-01632-9)
Supplement: Supplementary file 2 — Additional file 1: Figure S1. Effects of buffers and temperatures on the infectivity of spiked phages (A-C) and phage genomes (D-F). The percentages of phage activity (y-axis) or phage genomic recovery (y-axis, log10 scale) were determined by plaque assay at each different time point (x-axis) or qPCR. The error bars indicate the standard deviation with 3 replicates. Direct and indirect storage conditions were tested: in the direct storage condition, phage-spiked fecal samples were stored directly at 4 or − 80°C. In the indirect storage condition, phage-spiked fecal samples were first stored at 25°C for two days and then transferred to 4 or − 80°C, as described in the methods section. Figure S2. The effects of temperatures (A) and time (B) on the viral overall-alpha diversity with the measurement of Observed and Shannon index. NS indicates not significant, and two asterisks indicate a highly significant difference (p < 0.01, t-test). Figure S3. Representative of “sneaker contigs”. (A) The abundances of representative contigs annotated at different taxonomy levels, the selected contigs are based on contigs at high abundance compared to those in SM buffer (where the abundance was close to 0). (B) Genomic maps of the open reading frames (ORFs) which are predicted by prodigal and then annotated by blast to the NCBI protein database; the best hits were used to visualize the functional regions of these contigs. Different colors indicate different annotated proteins, directional boxes indicate ORFs in the respective orientation. NA: not assigned, HTP: hypothetical protein. Figure S4. Universal nuclease activity tests in the selected storage buffers (RNAlater, CANVAX and SM buffer). The selected buffers were spiked with exogenous DNA and then the activity of universal nuclease was tested. (A) The residues of exogenous DNA after 10 and 30 min treatments with universal nuclease in the selected buffers. (B) The residues of exogenous DNA in the selected buffers with a different [file 40168_2023_1632_MOESM1_ESM.docx]

**Supplemental Figures and Tables**

**Figure.S1** Effects of buffers and temperatures on the infectivity of spiked phages (A-C) and phage genomes (D-F). The percentages of phage activity (y-axis) or phage genomic recovery (y-axis, log10 scale) were determined by plaque assay at each different time point (x-axis) or qPCR. The error bars indicate the standard deviation with 3 replicates. Direct and indirect storage conditions were tested: in the direct storage condition, phage-spiked fecal samples were stored directly at 4 or − 80°C. In the indirect storage condition, phage-spiked fecal samples were first stored at 25°C for two days and then transferred to 4 or − 80°C, as described in the methods section.

**Figure.S2** The effects of temperatures (A) and time (B) on the viral overall-alpha diversity with the measurement of Observed and Shannon index. NS indicates not significant, and two asterisks indicate a highly significant difference (*p* < 0.01, t-test).

**Figure.S3** Representative of “sneaker contigs”. (A) The abundances of representative contigs annotated at different taxonomy levels, the selected contigs are based on contigs at high abundance compared to those in SM buffer (where the abundance was close to 0). (B) Genomic maps of the open reading frames (ORFs) which are predicted by prodigal and then annotated by blast to the NCBI protein database; the best hits were used to visualize the functional regions of these contigs. Different colors indicate different annotated proteins, directional boxes indicate ORFs in the respective orientation. NA: not assigned, HTP: hypothetical protein.

**Figure.S4** Universal nuclease activity tests in the selected storage buffers (RNAlater, CANVAX and SM buffer). The selected buffers were spiked with exogenous DNA and then the activity of universal nuclease was tested. (A) The residues of exogenous DNA after 10 and 30 min treatments with universal nuclease in the selected buffers. (B) The residues of exogenous DNA in the selected buffers with a different number of washes with SM buffer. The error bars indicate the standard deviation with 3 replicates.

**Fig. S1**


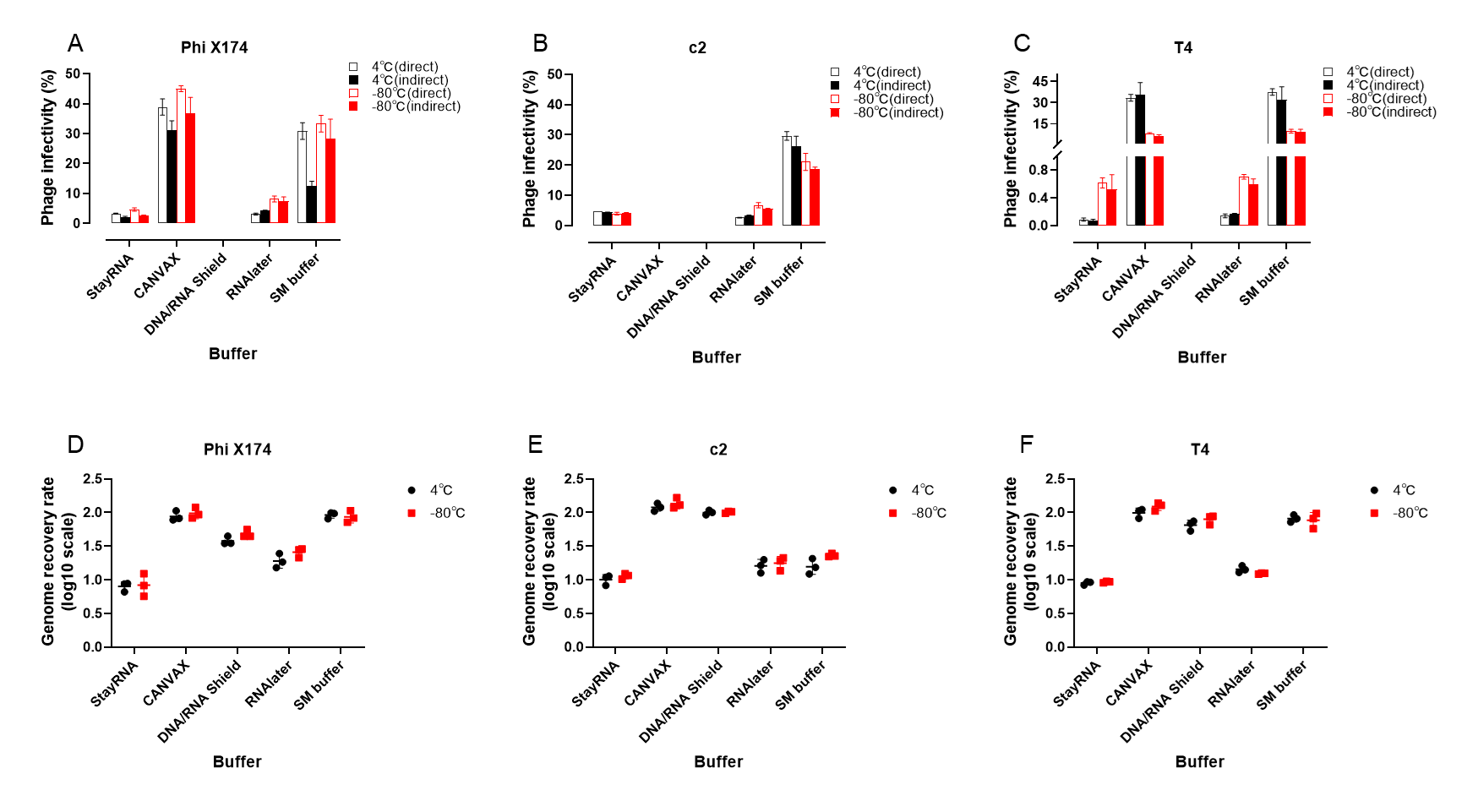


**Fig. S2**


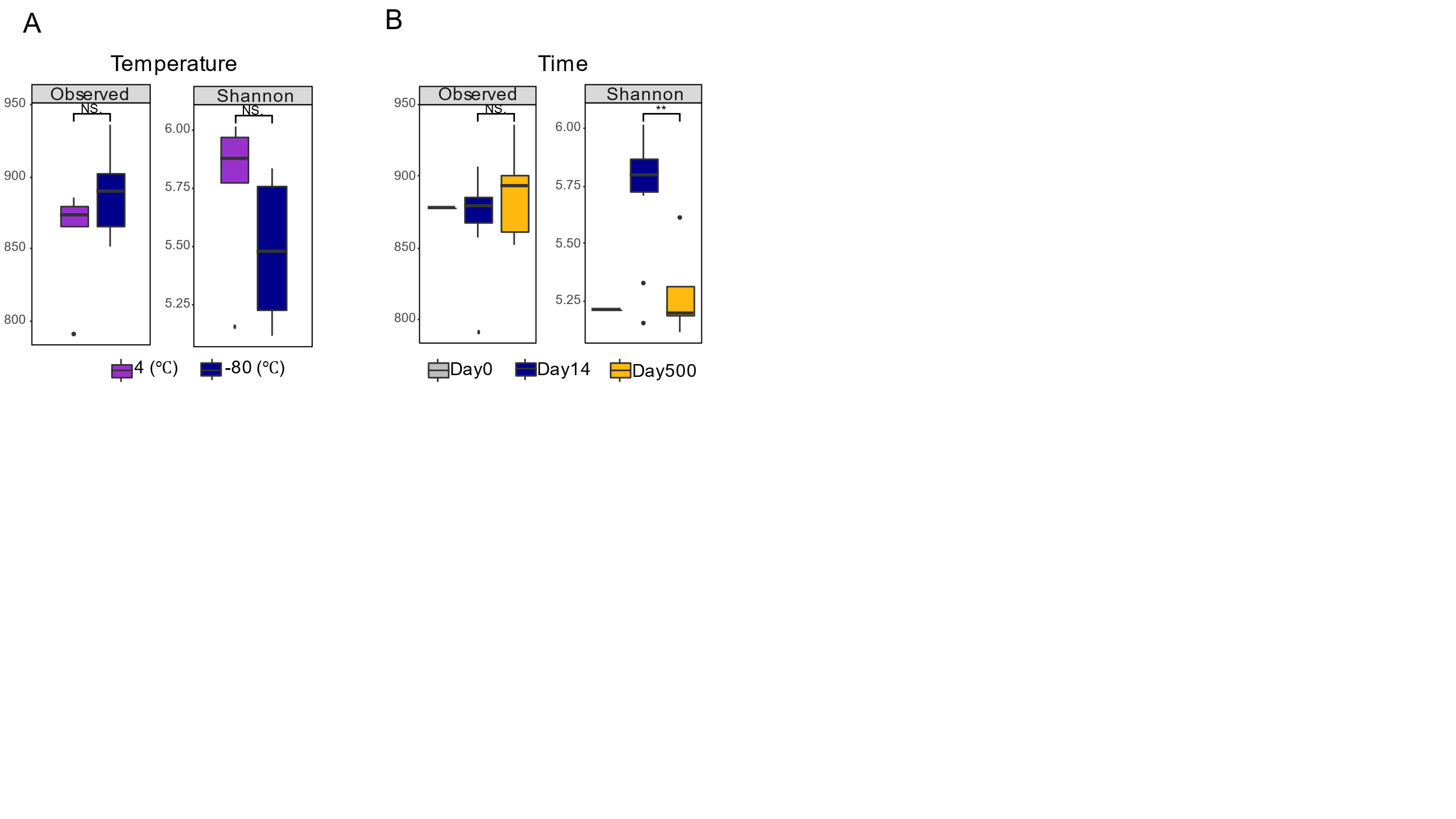


**Fig. S3**


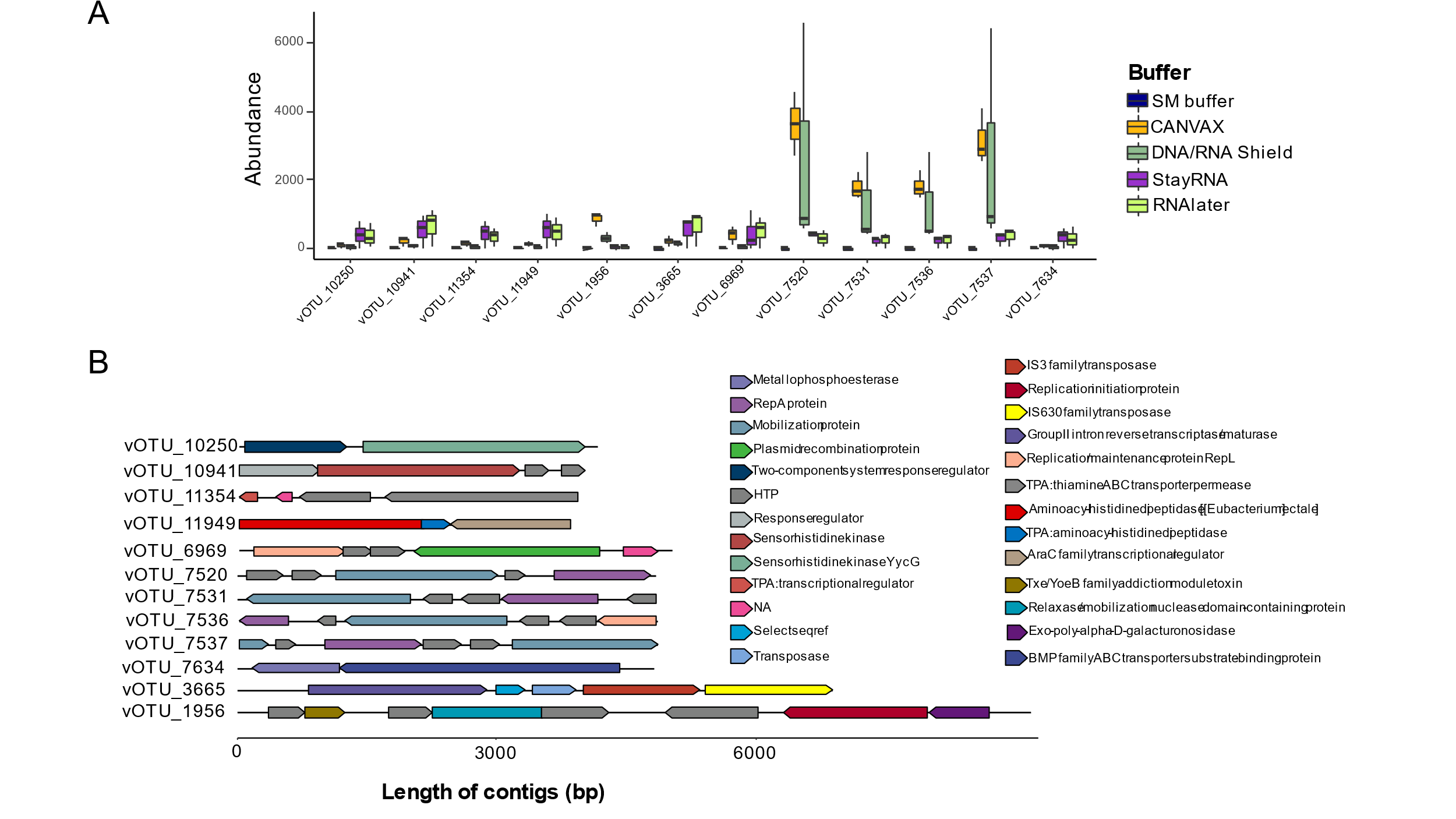


**Fig. S4**


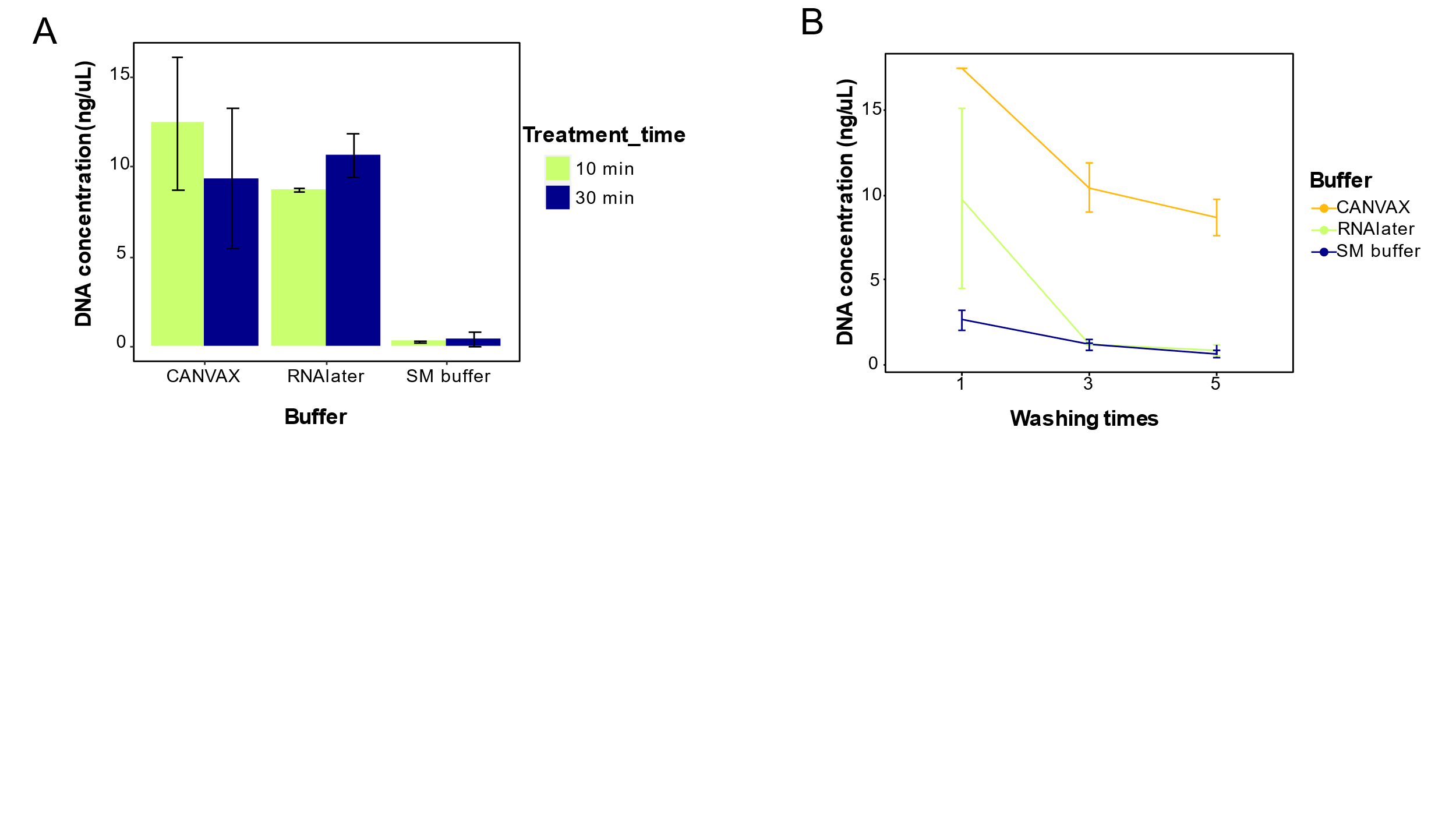


**Table S1** Plaque assay and qPCR-based phage recovery rate (%) after spiking phages (T4, c2 and Phi X174) in fresh fecal with different buffers (day0).

|  |  | StayRNA | CANVAX | DNA/RNA Shield | RNAlater | SM buffer |
| --- | --- | --- | --- | --- | --- | --- |
| Plaque  assay | T4 | 0.07 ± 0.008 | 26.0 ± 3.22 | ND | 0.35 ± 0 | 37.4 ± 3.51 |
|  | c2 | 17.0 ± 3.42 | ND | ND | 10.1 ± 1.42 | 39.7 ± 2.38 |
|  | Phi X174 | 3.36 ± 0.43 | 36.6 ± 1.34 | ND | 8.22 ± 2.13 | 34.6 ± 4.09 |
| qPCR | T4 | 7.98 ± 1.82 | 126.9 ± 27.6 | 78.6 ± 29.6 | 14.6 ± 3.72 | 74.7 ± 11.9 |
|  | c2 | 9.23 ± 2.92 | 170.2 ± 35.5 | 114.5 ± 10.9 | 19.9 ± 3.41 | 20.4 ± 6.97 |
|  | Phi X174 | 15.7 ± 3.45 | 163.0± 34.5 | 40.9 ± 5.38 | 28.5 ± 8.30 | 91.2 ± 7.06 |

ND: not detected

**Table S2** Differences in the fecal virome isolation process with different buffers

| Buffer | pH | Storage temperature (°C) | Storage time  (days) | Enrichment temperature (°C) | Final volume (μL) | Enrichment time (min) | DNA concentration (ng/μL) | Purity (260/280) |
| --- | --- | --- | --- | --- | --- | --- | --- | --- |
| Baseline | 7.66 | - | 0 | 4 | 280 | 180 | 6.22 | 2.69 |
| StayRNA | 4.84 | 4 | 14 | 4 | 140 | 120~180 | 6.00 | 2.56 |
|  |  | -80 | 14 |  | 280 |  | 5.65 | 2.66 |
|  |  | -80 | 500 |  | 140 |  | 4.64 | 2.51 |
| CANVAX | 7.46 | 4 | 14 | 25 | 300 | >300 | 51.0 | 2.09 |
|  |  | -80 | 14 |  | 420 |  | 42.0 | 2.09 |
|  |  | -80 | 500 |  | 280 |  | 40.6 | 2.06 |
| DNA/RNA Shield | 6.19 | 4 | 14 | 25 | 420 | 240~300 | 44.8 | 1.96 |
|  |  | -80 | 14 |  | 420 |  | 56.2 | 1.96 |
|  |  | -80 | 500 |  | 280 |  | 49.1 | 2.01 |
| RNAlater | 4.88 | 4 | 14 | 4 | 140 | 90~120 | 6. 41 | 2.74 |
|  |  | -80 | 14 |  | 140 |  | 5.86 | 2.78 |
|  |  | -80 | 500 |  | 140 |  | 4.63 | 2.63 |
| SM buffer | 7.66 | 4 | 14 | 4 | 280 | 180~210 | 7.3 | 2.71 |
|  |  | -80 | 14 |  | 140 |  | 6.62 | 2.73 |
|  |  | -80 | 500 |  | 280 |  | 6.32 | 2.62 |

**Table S3** Relative abundance (%) of viral composition at order level.

| Buffer | Storage temperature (°C) | Storage time (days) |  | Order_level (%) | | | | |
| --- | --- | --- | --- | --- | --- | --- | --- | --- |
|  |  |  | Caudovirales | | Herpesvirales | Petitvirales | Unclassified | Unknown |
| Baseline | - | 0 | 58.6 | | 0.04 | 0.04 | 31.2 | 10.0 |
| StayRNA | 4 | 14 | 33.3 | | 0.10 | 0.04 | 42.6 | 23.9 |
|  | -80 | 14 | 37.5 | | 0.12 | 0.10 | 40.1 | 22.2 |
|  | -80 | 500 | 56.8 | | 0.10 | 3.49 | 28.7 | 10.9 |
| CANVAX | 4 | 14 | 28.9 | | 0.06 | 0.45 | 33.4 | 37.1 |
|  | -80 | 14 | 29.7 | | 0.08 | 0.19 | 28.5 | 41.5 |
|  | -80 | 500 | 30.4 | | 0.17 | 0.26 | 21.8 | 47.3 |
| DNA/RNA Shield | 4 | 14 | 37.6 | | 0.19 | 0.00 | 37.7 | 24.5 |
|  | -80 | 14 | 37.9 | | 0.11 | 0.00 | 33.0 | 28.9 |
|  | -80 | 500 | 32.7 | | 0.14 | 0.01 | 20.3 | 46.8 |
| RNAlater | 4 | 14 | 31.7 | | 0.06 | 0.03 | 43.0 | 25.2 |
|  | -80 | 14 | 36.6 | | 0.10 | 0.08 | 39.5 | 23.7 |
|  | -80 | 500 | 56.2 | | 0.08 | 2.49 | 29.2 | 12.0 |
| SM buffer | 4 | 14 | 56.8 | | 0.06 | 3.61 | 30.1 | 9.41 |
|  | -80 | 14 | 57.0 | | 0.06 | 1.03 | 31.1 | 10.8 |
|  | -80 | 500 | 59.6 | | 0.02 | 3.04 | 27.4 | 9.93 |

NA: not assigned

**Table S4** Phages and their respective host bacteria in the present study.

| Phage (Family) | Shape | Nucleic acid | Host bacteria |
| --- | --- | --- | --- |
| Phi X174 (Microviridae) | Non-enveloped, isometric, cubic, icosahedral | Circular ssDNA | *Escherichia Coli* ATTC 13706 |
| c2 (Siphoviridae) | Non-enveloped, prolate-headed, long and non-contractile tail | Linear dsDNA | *Lactococcus lactis* MG 1363 |
| T4 (Myoviridae) | Non-enveloped, long and contractile tail, consisting of a sheath and a central tube | Linear dsDNA | *Escherichia Coli* DSM 613 |

**Table S5** Primers and targeted position for T4, c2 and Phi X174 genomes.

| Phage | Primers | Targeted position (bp) |
| --- | --- | --- |
| T4 | Forward: 5’-CACAGAGGAACGGTCTTGTAAA-3’ | 53,921 to 54,070 |
|  | Reverse: 5’-GAGAAGCCCTCCAGAATCATAAA-3’ |  |
| c2 | Forward: 5’-CGGATAGAACCACCAACAAGAG-3’ | 2,190 to 2,209 |
|  | Reverse: 5’-CTGGATGCCTAAAGACGAATGAG-3’ |  |
| Phi X174 | Forward: 5’-GAGAAGACGGTTACGCAGTT-3’ | 853 to 872 |
|  | Reverse: 5’-GCCTCTACGCGATTTCATAGT-3’ |  |

**Table S6** The number of reads and coverage of spiked phages (T4, c2 and Phi X174) to their respective reference genomes. The coverage rate was calculated by Botwie2.

| Buffer | Storage temperature (°C) | Storage time (days) | Total reads | Number of reads | | | Coverage | | | Mean depth | | |
| --- | --- | --- | --- | --- | --- | --- | --- | --- | --- | --- | --- | --- |
|  |  |  |  | C2 | Phi X174 | T4 | c2 | Phi X174 | T4 | c2 | Phi X174 | T4 |
| Baseline | - | 0 | 4191972 | 25154 | 511 | 18280 | 99.9 | 100 | 99.0 | 170 | 14.2 | 16.3 |
| StayRNA | 4 | 14 | 3350280 | 2299 | 156 | 451 | 98.5 | 96.0 | 26.2 | 15.6 | 4.36 | 0.40 |
|  | -80 | 14 | 4363504 | 7395 | 536 | 1935 | 99.9 | 99.5 | 63.4 | 50.0 | 14.9 | 1.71 |
|  | -80 | 500 | 3943580 | 18749 | 45139 | 6129 | 99.3 | 100 | 89.9 | 127 | 1259 | 5.44 |
| CANVAX | 4 | 14 | 3606452 | 320 | 424 | 209 | 72.0 | 98.9 | 14.5 | 2.20 | 11.9 | 0.19 |
|  | -80 | 14 | 2747160 | 258 | 201 | 85 | 70.8 | 99.5 | 5.9 | 1.75 | 5.61 | 0.07 |
|  | -80 | 500 | 2764752 | 440 | 578 | 161 | 85.0 | 100 | 10.8 | 2.99 | 16.1 | 0.14 |
| DNA/RNA Shield | 4 | 14 | 3344852 | 2531 | 2 | 1580 | 99.1 | 4.51 | 60.3 | 17.2 | 0.06 | 1.41 |
|  | -80 | 14 | 2685972 | 2051 | 40 | 1492 | 98.2 | 55.6 | 58.5 | 13.9 | 1.11 | 2.33 |
|  | -80 | 500 | 3265632 | 4267 | 292 | 4127 | 99.9 | 99.5 | 84.1 | 28.9 | 8.13 | 3.67 |
| RNAlater | 4 | 14 | 5394640 | 3249 | 273 | 1035 | 99.9 | 100 | 45.3 | 21.9 | 7.60 | 0.92 |
|  | -80 | 14 | 4772444 | 11537 | 386 | 1472 | 99.9 | 99.4 | 57.8 | 78.2 | 10.8 | 1.31 |
|  | -80 | 500 | 6529708 | 35451 | 75659 | 15333 | 98.9 | 100 | 98.5 | 240 | 2112 | 13.6 |
| SM buffer | 4 | 14 | 661636 | 8426 | 2302 | 11852 | 99.7 | 100 | 99.0 | 57.3 | 64.4 | 10.6 |
|  | -80 | 14 | 3086580 | 32379 | 22636 | 12277 | 99.8 | 100 | 97.2 | 219 | 632 | 10.9 |
|  | -80 | 500 | 3638596 | 30979 | 38846 | 9749 | 99.8 | 100 | 96.9 | 209 | 1084 | 8.67 |
